# Our Contemporaries

**Published:** 1916-08

**Authors:** 


### OUR CONTEMPORARIES

A story is told, sometimes in dialect, of a person who listened calmly to a torrent of abuse and then replied "All you have said I am, you are and then some." Editors who have expressed themselves on the subject of rolling manuscript and who roll their own journals before mailing, are requested to take notice.

---

The Medical Economist of 140 a, Floyd St., N. Y., is campaigning to eliminate the Boylan Law and asks the co-operation of the profession in signing a petition. While the intent of the law is excellent, it complicates professional duties, especially as the national Harrison law suffices and practically duplicates the safeguards against narcotic addiction, with numerous puzzling details of conflict.

---

Do you know that it is pleasanter to be well than sick?

That being comfortable is more agreeable than being uncomfortable?

That, for some of us, it is as hard to get money, when it is "easy" as when "tight?"

That toothache is unpleasant?

That bellyache unfits one, temporarily, for polite society?

That corns provoke profanity?

That fishermen are truthful but imaginative?

That all automobilists are not in the best society?

That health board men are not necessarily wiser than others?

T. W. in Homoeopathic Recorder. We heartily agree that this is quite as good as the majority of propagandist stuff that begins in the same way.

